# Diversity, Functional Complexity, and Translational Potential of Glial Cells in the Central Nervous System

**DOI:** 10.3390/ijms26189080

**Published:** 2025-09-18

**Authors:** Agata Wawrzyniak, Izabela Krawczyk-Marć, Agnieszka Żuryń, Jerzy Walocha, Krzysztof Balawender

**Affiliations:** 1 Department of Histology and Embryology, Faculty of Medicine, University of Rzeszów, 35-516 Rzeszow, Poland; awawrzyniak@ur.edu.pl (A.W.); izkrawczyk@ur.edu.pl (I.K.-M.); 2 Department of Histology and Embryology, Nicolaus Copernicus University in Toruń, 85-067 Bydgoszcz, Poland; azuryn@cm.umk.pl; 3 Department of Anatomy, Jagiellonian University Medical College, 31-008 Kraków, Poland; jwalocha@uj.edu.pl; 4 Department of Normal and Clinical Anatomy, Faculty of Medicine, University of Rzeszów, 35-516 Rzeszow, Poland

**Keywords:** astrocytes, oligodendrocytes, microglia, oligodendrocyte progenitor cells (NG2-glia), ependymocytes

## Abstract

Glial cells have emerged as active and dynamic regulators of central nervous system (CNS) function, far beyond their historically perceived supportive role. This review synthesizes the most recent advances in glial biology, highlighting novel molecular mechanisms, cutting-edge imaging methodologies, and translational strategies that redefine their role in health and disease. We emphasize new findings on astrocytic signaling in neurodegeneration, NG2-glia dynamics, and microglial modulation, providing forward-looking perspectives for glia-targeted therapeutic interventions. Recent breakthroughs in high-resolution in vivo imaging, single-cell transcriptomics, and gene-editing platforms are discussed in the context of their ability to unravel glial heterogeneity and functional plasticity. By integrating molecular insights with translational research, this review aims to bridge the gap between basic neuroscience and clinical applications, offering a framework for next-generation CNS therapies.

## 1. Introduction

**Figure 1:**
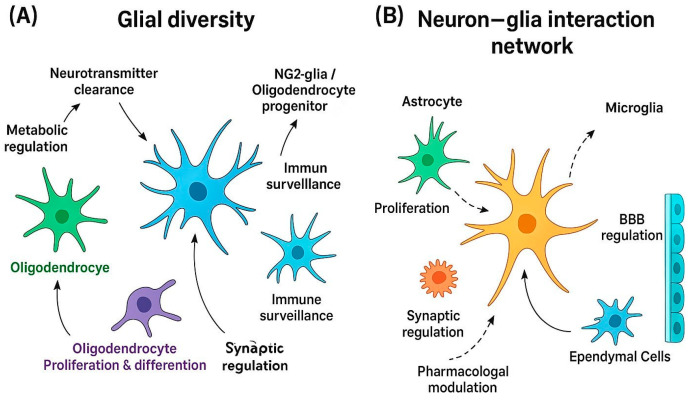
Integrated schematic overview of glial cell diversity and functional interactions with neurons in the CNS. (**A**) Glial diversity: astrocytes regulate neurotransmitter clearance, Ca^2+^ signaling, metabolic coupling, immune surveillance, and blood–brain barrier (BBB) integrity; oligodendrocytes provide myelin insulation and metabolic support; NG2-glia/oligodendrocyte progenitors contribute to proliferation, differentiation, and remyelination; microglia mediate immune surveillance, synaptic pruning, and inflammatory signaling; and ependymal cells regulate cerebrospinal fluid (CSF) dynamics and barrier function. (**B**) Neuron–glia interaction network: schematic representation of the functional interplay between neurons and glial cells. Solid arrows indicate established biological processes, while dashed arrows highlight therapeutic targets currently under investigation.

The CNS is composed not only of neurons, traditionally considered the primary units of information processing, but also of an equally crucial and diverse population of glial cells [1,2,3]. Once relegated to a merely supportive role, glial cells, including astrocytes, oligodendrocytes, and microglia in the CNS, as well as Schwann cells in the peripheral nervous system, are now acknowledged as dynamic regulators of neural function, actively shaping both the structural and functional architecture of the brain [1,4] (Figure 1A).

Groundbreaking advances in neurobiology have dismantled the long-standing neuron-centric paradigm, revealing glial cells as pivotal regulators of synaptic function, neurotransmission, metabolic homeostasis, immune defense, and neurovascular coupling [2,5]. These findings have profound clinical implications, as glial cell dysfunction has been implicated in a wide range of neurological and psychiatric disorders, including multiple sclerosis (MS), Alzheimer’s disease (AD), Parkinson’s disease (PD), Huntington’s disease (HD), schizophrenia, and depression [6,7,8]. Accordingly, glial cells are emerging as attractive therapeutic targets for both neuroprotective interventions and regenerative medicine approaches. Glial cells not only outnumber neurons but also exhibit remarkable phenotypic and functional heterogeneity, with region-specific specializations and context-dependent behaviors. They form intricate networks with neurons and blood vessels and contribute to essential homeostatic functions, including extracellular ion buffering, neurotransmitter clearance, synapse formation and elimination, immune modulation, and myelination. This dynamic interplay enables plastic responses to injury, stress, and disease, underpinning many forms of neural adaptation. Understanding glial biology is now recognized as a prerequisite for deciphering the complexity of CNS function and dysfunction. Their regulatory roles extend beyond neurodevelopment into adulthood, modulating plasticity and resilience under both physiological and pathological conditions. Importantly, glial cells are not only involved in disease progression but also in its resolution, offering opportunities for innovative glia-targeted therapies. This review provides a comprehensive synthesis of glial biology, integrating recent mechanistic insights with translational perspectives. We highlight cutting-edge developments, such as single-cell transcriptomics for subtype characterization, in vivo imaging of dynamic neuron–glia interactions, and gene-editing strategies aimed at modulating glial function. Particular attention is given to underexplored cell populations (e.g., NG2-glia, ependymal cells) and to emerging therapeutic concepts, including microglia reprogramming and astrocyte-targeted metabolic interventions. By linking fundamental discoveries to clinical applications, this article aims to offer a forward-looking framework for next-generation glia-based diagnostics and treatments [6,9,10].

## 2. Glial Cells in Neuronal Network Function

The CNS comprises a highly intricate and dynamic network of neurons and glial cells. Historically perceived as passive support elements, glial cells are now recognized as active modulators of neuronal network function [4,9]. Although neurons have long been considered the principal signaling units of the nervous system, it is increasingly evident that glia play indispensable roles in regulating synaptic activity, neurogenesis, homeostasis, and immune surveillance. Glial cells encompass several specialized subtypes, including astrocytes, oligodendrocytes, microglia, and NG2-glia, each contributing uniquely to the structural and functional integrity of the CNS [3,9] (Figure 1B).

Astrocytes maintain ionic and neurotransmitter homeostasis and modulate synaptic transmission; oligodendrocytes generate myelin and support axonal metabolism; NG2-glia serve as proliferative precursors capable of responding to injury; and microglia act as the primary immune effectors of the CNS [1,10,11,12,13,14]. Despite their essential functions, glial cells have traditionally received less attention than neurons, due in part to the technical challenges associated with studying these heterogeneous populations [9,10]. However, advances in high-resolution imaging, genetic lineage tracing, and transcriptomic profiling have significantly enhanced our understanding of glial cell biology and their contributions to CNS physiology and disease [15,16,17]. Incorporating glial cells into models of neuronal networks reveals an additional layer of complexity. Unlike the classical neuron-centric models, neuron–glia interactions involve bidirectional signaling mechanisms, including calcium-based excitability, gliotransmission, and metabolic coupling. This expanded connectivity increases not only the number of cells involved but also the diversity of functional interactions across neural circuits [3,14]. From a developmental perspective, CNS macroglia, including astrocytes, oligodendrocytes, and NG2-glia, originate from neural stem cells, whereas microglia derive from primitive macrophages in the yolk sac that colonize the CNS during early embryogenesis [12,13,18]. Microglia remain the primary immunocompetent cells in the adult CNS, contributing to synaptic remodeling, pathogen defense, and injury response. Glial dysfunction has been implicated in a wide range of neurological and neurodegenerative disorders, including AD, MS, epilepsy, and psychiatric conditions [1,10,14]. The pathological contributions of each glial subtype are context-dependent and can involve both loss-of-function mechanisms (e.g., impaired glutamate clearance by astrocytes) and gain-of-toxic-function mechanisms (e.g., chronic microglial activation via NF-κB). Understanding these distinct roles is critical for elucidating disease mechanisms and developing glia-targeted therapeutic strategies [1,10,14].

### 2.1. Astrocytes

Astrocytes are the most abundant and one of the most morphologically diverse glial cell populations in the CNS, exerting critical influence over brain function and homeostasis [19,20,21]. Their structural and functional heterogeneity enables region-specific adaptations and context-dependent responses to physiological and pathological cues. Historically regarded as passive support cells, astrocytes are now recognized as multifunctional hubs that regulate virtually every aspect of neural activity [22]. They are engaged in synaptic modulation, metabolic and trophic support, maintenance of the blood–brain barrier (BBB), neurovascular coupling, immune surveillance, interstitial fluid regulation, and tissue repair. This broad functional repertoire places astrocytes at the center of modern neurobiological research, particularly in the context of neuroprotection, regenerative therapies, and precision glia-targeted interventions [21,22].

#### 2.1.1. Structural, Metabolic, and Energetic Support

Astrocytes provide a dynamic structural framework for the CNS, anchoring neuronal processes to the vasculature and preserving the extracellular ionic milieu essential for high-fidelity synaptic transmission [21,23]. They tightly regulate potassium, calcium, and neurotransmitter concentrations (including glutamate and GABA) within the synaptic cleft, preventing excitotoxicity and ensuring temporal precision of neuronal signaling [20,24]. Functioning as metabolic intermediaries, astrocytes form a pivotal interface between the circulatory system and neural tissue. Through their perivascular endfeet, enriched in aquaporin-4 (AQP4) channels, they facilitate glucose uptake from capillaries and convert it to lactate via aerobic glycolysis. This lactate is then delivered to neurons through the astrocyte–neuron lactate shuttle (ANLS), primarily mediated by monocarboxylate transporters MCT1 and MCT4, serving as a rapidly mobilizable energy substrate during heightened synaptic activity, long-term potentiation (LTP), and memory consolidation [25,26,27]. In addition to metabolic fueling, astrocytes actively participate in the glutamate–glutamine cycle via excitatory amino acid transporters (EAAT1/GLAST and EAAT2/GLT-1), thereby terminating excitatory transmission and maintaining glutamatergic balance. This neurotransmitter recycling is vital not only for signal fidelity but also for preventing neurotoxicity under conditions of metabolic stress or injury [20].

#### 2.1.2. Synaptic Regulation and Plasticity

Astrocytes are indispensable components of the tripartite synapse, ensheathing pre- and postsynaptic terminals to sense and respond to neuronal activity [28,29]. They modulate synaptic efficacy by controlling extracellular neurotransmitter clearance and by releasing gliotransmitters such as ATP, D-serine, and glutamate through calcium-dependent exocytosis or hemichannel-mediated pathways. These gliotransmitters regulate neuronal excitability, modulate N-methyl-D-aspartate receptor (NMDAR) function, and influence LTP and long-term depression (LTD) [29,30,31]. Astrocytic signaling is bidirectional: synaptic activity triggers intracellular Ca^2+^ waves in astrocytes, which in turn modulate local synaptic networks. During development, astrocytes secrete synaptogenic molecules such as thrombospondins, hevin, and SPARC, promoting synapse formation. In the mature CNS, they contribute to synaptic pruning by interacting with complement proteins (C1q, C3) and microglia. Through these mechanisms, astrocytes coordinate both the construction and refinement of neural circuits, positioning them as active architects of network plasticity.

#### 2.1.3. Regulation of Blood Flow and Neurovascular Coupling

Astrocytes play a pivotal role in neurovascular coupling, translating neuronal activity into rapid and spatially precise vascular responses that match local metabolic demands [23]. Synaptic activation triggers intracellular Ca^2+^ transients in astrocytic endfeet, which stimulate the release of vasoactive mediators, including prostaglandins (e.g., PGE_2_), nitric oxide (NO), and epoxyeicosatrienoic acids (EETs) [24]. These molecules act on smooth muscle cells of arterioles and pericytes, inducing vasodilation in adjacent microvessels. In some contexts, astrocytes can also evoke vasoconstriction via arachidonic acid metabolites such as 20-HETE, demonstrating bidirectional vascular regulation. This mechanism ensures timely delivery of oxygen and glucose to active brain regions, preserves neuronal excitability, and prevents hypoxia-related damage [32]. Disruption of astrocyte-mediated neurovascular coupling has been implicated in AD, small vessel disease, and ischemic stroke, highlighting its translational relevance as a therapeutic target.

Specifically in Alzheimer’s disease, impaired Ca^2+^ signaling in astrocytic endfeet and mislocalization of aquaporin-4 (AQP4) channels disrupt glymphatic clearance, thereby promoting amyloid-β accumulation and vascular dysfunction [19,22,33].

In the context of cerebral small vessel disease, astrocytic dysfunction compromises perivascular potassium buffering and endfoot polarization, exacerbating vascular stiffness and impairing microcirculation [34,35].

During ischemic stroke, excessive release of vasoactive gliotransmitters such as ATP and glutamate, together with dysregulated Ca^2+^ waves, facilitates spreading depolarizations, aggravating blood–brain barrier breakdown and infarct progression [36,37].

#### 2.1.4. Blood–Brain Barrier Maintenance and Neuroprotection

Astrocytes contribute substantially to the formation, maturation, and dynamic regulation of the BBB through their perivascular endfeet, which ensheath cerebral capillaries and closely interact with endothelial cells and pericytes [23,33]. They secrete signaling molecules such as angiopoietin-1, sonic hedgehog (Shh), and transforming growth factor-β (TGF-β), which enhance tight junction integrity and suppress vesicular transcytosis [34]. This selective barrier prevents the entry of neurotoxic substances while allowing regulated passage of essential metabolites, amino acids, and hormones. Beyond their barrier function, astrocytes exert neuroprotective effects by releasing brain-derived neurotrophic factor (BDNF), nerve growth factor (NGF), antioxidant molecules (e.g., glutathione), and anti-inflammatory cytokines [35]. Following CNS injury, astrocytes undergo reactive astrogliosis—a state characterized by cellular hypertrophy, upregulation of glial fibrillary acidic protein (GFAP), cytoskeletal remodeling, and transcriptomic reprogramming. Reactive astrocytes form a glial scar that spatially isolates damaged tissue and initiates a cascade of repair mechanisms, including axonal growth inhibition and extracellular matrix remodeling [33,34]. While glial scarring limits the spread of injury, excessive or prolonged scar formation can impede axonal regeneration, making it a double-edged process with both beneficial and detrimental outcomes.

#### 2.1.5. Inflammatory Modulation and Immune Signaling

Astrocytes are central orchestrators of CNS immune responses, capable of sensing and integrating inflammatory cues via pattern recognition receptors (PRRs) and cytokine receptors [35]. Upon activation, they produce a wide range of cytokines (e.g., IL-1β, IL-6, TNF-α), chemokines (e.g., CCL2/MCP-1, CXCL10), and lipid mediators, which influence microglial activation states, BBB permeability, and recruitment of peripheral immune cells [3,36]. In acute injury, astrocyte-derived signals often promote neuroprotection by limiting inflammatory spread, enhancing debris clearance, and supporting tissue repair. However, chronic or dysregulated activation—frequently associated with persistent NF-κB or JAK/STAT3 signaling—can drive maladaptive neuroinflammation, glial scarring, and neuronal dysfunction [37]. This dual role, shifting between neuroprotective and neurotoxic phenotypes, underscores the context-dependent nature of astrocytic responses in health and disease. Dysregulated astrocytic inflammation is increasingly recognized as a pathogenic contributor in MS, AD, ALS, and traumatic CNS injury [36,38]. Modulating astrocytic immune functions therefore represents a promising avenue for therapeutic intervention in a wide spectrum of neurological disorders.

#### 2.1.6. Involvement in Neurological Disorders

Astrocytic dysfunction is increasingly recognized as a central driver in the pathogenesis and progression of numerous neurodegenerative disorders, including AD, PD, ALS, HD, and chronic traumatic encephalopathy (CTE) [35,39]. Recent high-resolution imaging and single-cell transcriptomic analyses have revealed that astrocytes undergo profound phenotypic and molecular reprogramming in these conditions, often shifting towards reactive states (A1 neurotoxic or A2 neuroprotective phenotypes) that profoundly influence neuronal survival and network stability [22].

In Alzheimer’s disease (AD), astrocytic pathology is characterized by dysregulated calcium signaling, impaired glutamate clearance due to downregulation and dysfunction of excitatory amino acid transporter 2 (EAAT2/GLT-1), and mislocalization of aquaporin-4 (AQP4) channels in perivascular endfeet, leading to glymphatic system impairment and neurovascular uncoupling [40,41,42]. Moreover, impaired mitochondrial fatty acid metabolism in astrocytes has been linked to chronic neuroinflammation and neurodegeneration [43]. Recent studies further highlight the role of astrocytic apolipoprotein E (ApoE) isoforms in amyloid-β aggregation and clearance, as well as the contribution of reactive astrocytes to tau hyperphosphorylation and propagation [22,44]. These alterations collectively promote excitotoxicity, impaired amyloid-β clearance, chronic neuroinflammation, and synaptic dysfunction. The balance between A1 (neurotoxic) and A2 (neuroprotective) astrocytic phenotypes appears critical in determining neuronal vulnerability in AD [38,39,45,46,47]. Importantly, elevated levels of astrocyte-derived GFAP in plasma and CSF are now recognized as highly sensitive biomarkers of astrocytic injury and early AD progression, supporting their diagnostic and prognostic utility [48,49]. In addition, AQP4-IgG autoantibodies serve as specific diagnostic markers for neuromyelitis optica spectrum disorders, further underscoring the translational relevance of astrocytic endfeet dysfunction.

In Parkinson’s disease (PD), astrocytic mitochondrial dysfunction—linked to impaired PGC-1α signaling and reduced Nrf2-mediated antioxidant defense—exacerbates oxidative stress and accelerates dopaminergic neuron loss in the substantia nigra. Emerging evidence indicates that reactive astrocytes in PD not only fail to buffer oxidative stress but also modulate α-synuclein propagation, amplifying neurodegeneration [50]. Astrocytes also fail to maintain adequate levels of glutathione and metabolic substrates (e.g., lactate), further compromising dopaminergic neuron survival. In addition, astrocytic dysfunction has been implicated in the impaired clearance of α-synuclein aggregates, which contributes to progressive neurodegeneration. Recent work demonstrated imbalanced mitochondrial dynamics in both human PD tissue and α-synuclein mouse models [51]. In parallel, astrocytes have been shown to exert protective effects by modulating α-synuclein aggregation, suggesting therapeutic potential in PD [52]. Dysregulated secretion of neurotrophic factors, such as GDNF and BDNF, further limits the ability of astrocytes to support dopaminergic neurons and synaptic integrity [53,54,55,56,57,58,59].

In amyotrophic lateral sclerosis (ALS), astrocytic deficits compromise neuronal survival through metabolic dysregulation. Reduced lactate delivery via monocarboxylate transporters (MCT1, MCT4) and impaired glutamate homeostasis drive excitotoxicity and accelerate motor neuron degeneration. Divergent single-cell transcriptomic and epigenomic alterations in ALS and FTD patients with C9orf72 mutations have revealed profound changes in astrocytic support functions [60], underscoring their contribution to disease progression.

In Huntington’s disease (HD) and chronic traumatic encephalopathy (CTE), reactive astrocytes contribute to neuronal dysfunction not only by altering neurotransmitter and ion homeostasis but also through aberrant protein secretion. Recent studies have demonstrated that astrocyte-derived proteins disrupt neuronal development and synaptic stability in disease models [61], further underscoring the pathogenic potential of reactive astrocytosis.

**Figure 2:**
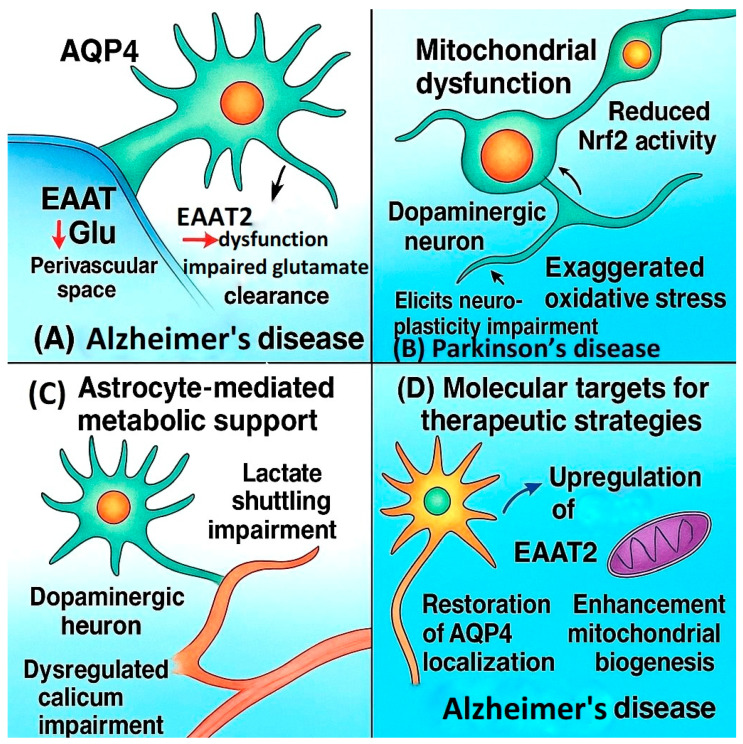
Astrocytic dysfunction across neurodegenerative diseases. (**A**) In Alzheimer’s disease, EAAT2 dysfunction impairs glutamate uptake, contributing to excitotoxicity, alongside dysregulated calcium signaling and mislocalization of AQP4 channels. (**B**) In Parkinson’s disease, mitochondrial dysfunction and reduced Nrf2 activity exacerbate oxidative stress and dopaminergic neuron loss. (**C**) In amyotrophic lateral sclerosis (ALS), impaired lactate shuttling reduces metabolic support for motor neurons. (**D**) Potential therapeutic strategies include upregulation of EAAT2, restoration of AQP4 localization, and enhancement of mitochondrial biogenesis.

Collectively, these findings demonstrate that astrocytic dysfunction extends far beyond passive support and is intimately involved in the pathophysiology of multiple neurological diseases. These mechanistic insights have revealed multiple therapeutic entry points, including upregulation of EAAT2 expression, restoration of AQP4 polarity, enhancement of mitochondrial biogenesis, and modulation of inflammatory signaling pathways such as NF-κB, JAK/STAT3, and Nrf2. Astrocytic alterations extend beyond neurotransmitter clearance and metabolic support; they also involve disruption of ionic homeostasis, compromised BBB maintenance, aberrant cytokine release, and maladaptive crosstalk with microglia and oligodendrocytes. Given their multifaceted contributions to CNS pathology, astrocytes are now considered both biomarkers and therapeutic targets. Translating these insights into clinical interventions will require integrative approaches combining molecular profiling, advanced imaging, and targeted pharmacological or gene therapy strategies aimed at restoring astrocytic homeostasis and functions [22] (Figure 2). Astrocyte aging has emerged as a critical determinant of CNS vulnerability, with recent work demonstrating progressive impairment of metabolic support and neuroprotective functions in aged astrocytes [62].

### 2.2. Oligodendrocytes

Oligodendrocytes are highly specialized myelinating glial cells of the CNS, essential for both neural physiology and the long-term stability of neuronal networks [45]. Historically viewed primarily as structural insulators, they are now recognized as dynamic, metabolically active cells that integrate neuronal activity patterns, adapt their myelinating behavior accordingly, and contribute to the fine-tuning of circuit function [45,46]. Dysregulation of oligodendrocyte function has been implicated not only in classical demyelinating diseases, such as MS and leukodystrophies, but also in neurodegenerative disorders, neurodevelopmental conditions, and psychiatric diseases including schizophrenia and major depressive disorder [45,46].

#### 2.2.1. Myelination and Neural Transmission Efficiency

Oligodendrocytes produce compact, multilamellar myelin sheaths in lipid-rich membranes that ensheath axons in discrete segments separated by nodes of Ranvier [47,53,54]. This configuration enables saltatory conduction, whereby action potentials propagate rapidly between nodes, achieving conduction velocities up to 100-fold greater than in unmyelinated fibers while conserving metabolic energy. Each oligodendrocyte extends numerous processes, myelinating multiple axons simultaneously, thereby maximizing efficiency across large neural territories [53,54]. Recent advances in in vivo imaging have demonstrated that myelination is not a fixed developmental endpoint, but an activity-dependent, plastic process that persists into adulthood. Experience-driven remodeling of myelin sheaths fine-tunes conduction timing across distributed networks, contributing to motor learning, sensory integration, and cognitive flexibility. Disruption of this plasticity may underlie certain forms of cognitive decline and maladaptive network activity seen in disease [47,54].

#### 2.2.2. Metabolic and Structural Support

Beyond electrical insulation, oligodendrocytes serve as metabolic lifelines for axons. They deliver lactate and pyruvate via monocarboxylate transporters (MCT1, MCT2) to sustain axonal ATP production, particularly during periods of high-frequency firing or metabolic stress [53]. This metabolic coupling preserves ionic gradients, prevents conduction failure, and supports long-term axonal survival [42,43,44,45,46,47,50,51,52,53,54,55,56]. They also play a role in redox regulation, supplying glutathione and other antioxidant molecules to neutralize reactive oxygen species (ROS), which are especially damaging to lipid-rich myelin membranes. Structurally, oligodendrocytes determine axonal caliber and regulate the molecular organization of nodes of Ranvier, ensuring optimal clustering of voltage-gated sodium and potassium channels [45,46,47,63,64]. These interactions stabilize axonal function and protect against degeneration in aging and disease.

#### 2.2.3. Translational Relevance

Oligodendrocyte biology has emerged as a therapeutic frontier. Enhancing oligodendrocyte survival, differentiation, and remyelination holds promise for treating MS, traumatic brain injury, and even certain neuropsychiatric conditions. Small molecules, growth factors (e.g., IGF-1, BDNF), and modulation of intracellular signaling pathways (e.g., PI3K–Akt–mTOR, ERK1/2) are under active investigation. Recent translational work has highlighted compounds such as clemastine fumarate that promote remyelination in MS patients, as well as gene therapy approaches designed to enhance OPC differentiation [65,66]. Moreover, recognition of oligodendrocyte metabolic support as a determinant of axonal survival underscores new strategies aimed at improving bioenergetic resilience in white matter tracts, with translational implications for both demyelinating and neurodegenerative disorders [65,66].

#### 2.2.4. Axonal Maintenance and Repair

Beyond sustaining intact axons, oligodendrocytes actively participate in the repair, remodeling, and long-term stabilization of injured neuronal circuits. Following CNS injury, they contribute to the clearance of myelin debris—either directly or via signaling to microglia to create a permissive environment for regeneration [67,68,69]. Oligodendrocyte progenitor cells (OPCs), which remain widely distributed in the adult CNS, can proliferate and differentiate into mature, myelinating oligodendrocytes in response to demyelination. This process is critical for restoring conduction velocity and functional connectivity after injury [67,68,69]. Mature oligodendrocytes also secrete growth-promoting and trophic factors, including insulin-like growth factor 1 (IGF-1) and fibroblast growth factor 2 (FGF2), which facilitate axonal regeneration, promote cytoskeletal stabilization, and support synaptic reorganization. This dual capacity—for both structural replacement and biochemical suport—positions oligodendrocytes as central players in white matter repair strategies [67,68,69].

#### 2.2.5. Modulation of Neuronal Activity

Accumulating evidence demonstrates that oligodendrocytes modulate neuronal activity through mechanisms extending beyond myelination. They release brain-derived neurotrophic factor (BDNF), neuregulins, and other neuromodulatory molecules that influence synaptic efficacy, firing patterns, and network excitability [55,70]. This biochemical signaling operates in parallel with conduction-speed regulation, providing a multidimensional influence on information processing. Moreover, crosstalk between oligodendrocytes and interneurons can adjust inhibitory tone, further refining circuit dynamics [58]. These interactions highlight the role of oligodendrocytes as active participants in activity-dependent synaptic plasticity.

#### 2.2.6. Role in CNS Plasticity and Learning

Recent studies challenge the traditional view that learning and memory are exclusively neuron-driven processes. Oligodendrocyte activity, particularly activity-regulated myelination, modulates conduction velocity along specific axonal pathways, thereby fine-tuning temporal coordination between neuronal ensembles [70,71,72]. This temporal precision is essential for synchronous oscillatory activity underlying complex behaviors. Experimental paradigms in rodents have demonstrated that engagement in novel motor or cognitive tasks induces measurable changes in myelin thickness and internodal length, supporting the concept that myelin is a dynamic substrate for experience-dependent plasticity [71,72,73]. Disruption of this adaptive myelination has been linked to impairments in working memory, decision-making, and sensorimotor integration.

#### 2.2.7. Involvement in Neurological and Psychiatric Disorders

Oligodendrocyte pathology is a hallmark of a broad spectrum of neurological diseases. In MS, autoimmune-mediated destruction of myelin and oligodendrocytes results in conduction block, axonal degeneration, and progressive disability [65,74,75]. However, oligodendrocyte involvement extends beyond classical demyelinating diseases. Transcriptomic and histopathological studies have identified altered expression of myelin-related genes, reduced oligodendrocyte density, and aberrant myelin ultra-structure in psychiatric disorders such as schizophrenia, bipolar disorder, and major depressive disorder [65,76,77]. These abnormalities may disrupt white matter connectivity and network synchrony, contributing to cognitive and affective symptoms.

**Figure 3:**
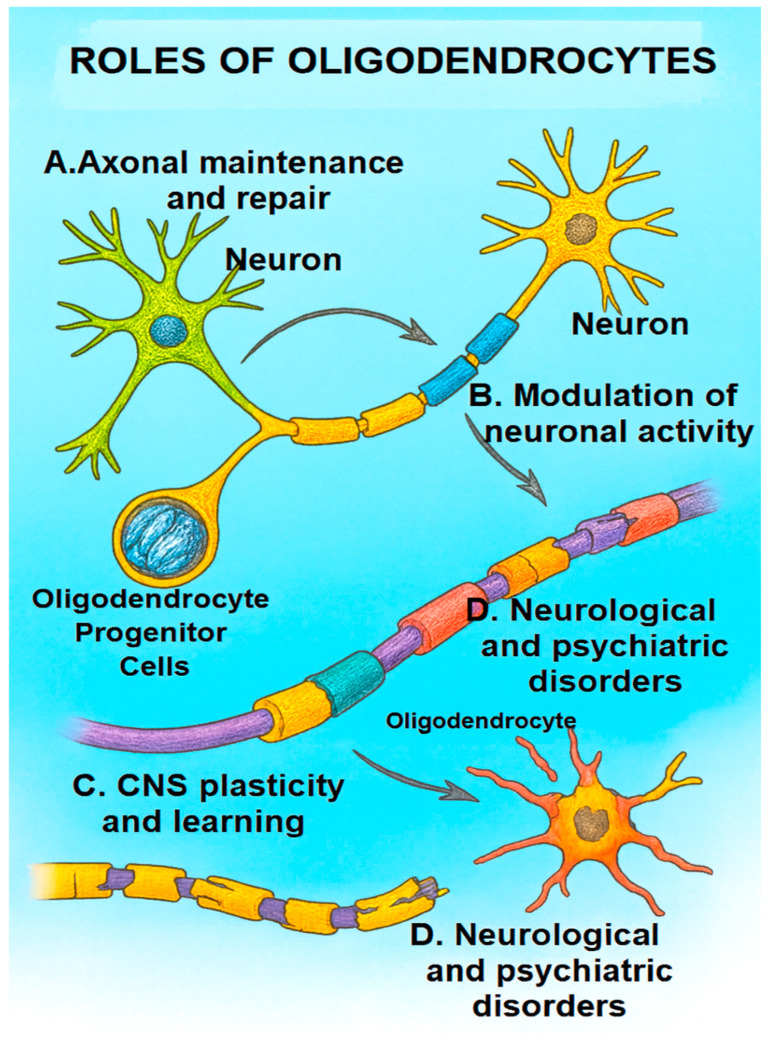
Roles of oligodendrocytes in the CNS. (A) Axonal maintenance and repair—OPCs differentiate into mature oligodendrocytes, secrete growth factors, and support axonal regeneration. (B) Modulation of neuronal activity—release of neurotrophic and modulatory factors (e.g., BDNF, neuregulins) influencing synaptic strength, firing patterns, and network excitability. (C) CNS plasticity and learning—activity-dependent myelin remodeling fine-tunes conduction velocity and neuronal synchrony, contributing to motor and cognitive learning. (D) Neurological and psychiatric disorders—oligodendrocyte dysfunction and myelin abnormalities contribute to MS, schizophrenia, and major depressive disorder.

Additionally, oligodendrocyte dysfunction has been implicated in neurodegenerative disorders—including AD and HD where impaired metabolic support and remyelination exacerbate neuronal vulnerability. Given their multifaceted roles, therapeutic approaches aimed at protecting, regenerating, or functionally modulating oligodendrocytes are being actively pursued. Such strategies include promoting OPC differentiation, enhancing mitochondrial resilience, and modulating immune-mediated myelin injury—interventions that hold potential for both neurological and psychiatric disease management [65,74,75,76,77]. Importantly, these findings underscore that oligodendrocyte pathology extends beyond classical demyelinating diseases and encompasses a wide spectrum of neurodegenerative, developmental, and psychiatric conditions, including multiple sclerosis, schizophrenia, and major depressive disorder (Figure 3).

Myelin basic protein (MBP) concentrations in CSF provide a reliable biomarker of demyelination and correlate with disease severity in multiple sclerosis [78]. Furthermore, MOG-IgG autoantibodies define MOG-associated disorders (MOGAD) as a distinct nosological entity from MS and NMOSD [79,80,81,82].

In addition, autoantibody-mediated disorders such as MOG antibody-associated disease (MOGAD) and neuromyelitis optica spectrum disorders (NMOSD) further illustrate the central role of glial pathology. In MOGAD, autoantibodies directed against myelin oligodendrocyte glycoprotein (MOG) induce demyelination, oligodendrocyte dysfunction, and secondary astrocytic reactivity [83,84]. In NMOSD, aquaporin-4 (AQP4) autoantibodies selectively target astrocytic endfeet, leading to complement-mediated astrocyte injury, blood–brain barrier disruption, and subsequent secondary demyelination [85,86]. These disorders underscore the translational importance of targeting astrocytic and oligodendrocytic antigens in antibody-driven CNS pathology, and highlight the therapeutic relevance of immunomodulatory strategies such as anti-CD20 therapies and complement inhibition.

### 2.3. NG2-Glia and Microglia: Versatile Regulators of CNS Homeostasis and Plasticity

NG2-glia and microglia represent two highly dynamic and functionally versatile glial populations that are indispensable for CNS development, homeostasis, and repair. Despite their distinct developmental origins NG2-glia arising from neuroectodermal lineage as progenitors of myelinating oligodendrocytes, and microglia deriving from yolk sac-born primitive macrophages as the resident immune cells of the CNS-emerging evidence underscores a substantial degree of functional convergence. Both cell types engage in neuroplasticity, injury response, immune regulation, and synaptic remodeling, reflecting their integrated roles in neural network maintenance and adaptation [87,88].

#### 2.3.1. Developmental and Reparative Functions

NG2-glia are the primary source of new oligodendrocytes during both developmental myelination and adult CNS plasticity, enabling activity-dependent adaptive myelination that fine-tunes conduction velocity and synchrony across neuronal networks [89,90,91]. Following CNS injury, NG2-glia proliferate, migrate toward demyelinated lesions, and differentiate into mature myelinating cells, thereby facilitating remyelination and functional recovery [91,92,93]. Microglia, in parallel, contribute to developmental wiring of the brain through activity-dependent synaptic pruning, selectively eliminating redundant synapses to optimize circuit efficiency [94]. They also regulate adult neurogenesis, particularly in the hippocampal dentate gyrus, via secretion of cytokines, chemokines, and neurotrophic factors that influence neural stem/progenitor proliferation, differentiation, and integration [95,96].

#### 2.3.2. Synaptic Modulation and Neuroplasticity

NG2-glia possess a unique capacity to form synapse-like junctions with neurons and express functional neurotransmitter receptors, including AMPA-type glutamate receptors and GABA-A receptors. This synaptic connectivity enables them to directly sense neuronal activity, modulate their own proliferation and differentiation, and influence local synaptic plasticity [93]. Microglia modulate synaptic strength and connectivity through the release of cytokines, complement proteins, and neurotrophic factors that shape both excitatory and inhibitory neurotransmission [97]. Their highly motile processes continuously survey the CNS microenvironment, enabling rapid morphological and functional adaptations to neuronal activity changes or tissue injury [97,98]. This bidirectional neuron–microglia communication contributes to both short-term synaptic tuning and long-term structural remodeling of neural circuits.

#### 2.3.3. Immune Surveillance and Inflammatory Responses

Microglia are the principal immune sentinels of the CNS, maintaining continuous surveillance for pathogens, injury signals, and homeostatic perturbations. Upon activation, they undergo phenotypic transformation into phagocytic, antigen-presenting cells, releasing a broad spectrum of inflammatory mediators—including interleukins, chemokines, and reactive oxygen/nitrogen species—that coordinate the innate immune response [99,100,101,102,103]. While acute microglial activation is essential for tissue defense and repair, chronic overactivation drives neuroinflammation, oxidative stress, and bystander neuronal injury, thereby contributing to neurodegenerative disease progression [101,103,104,105]. Microglia have attracted increasing attention as translational targets due to their central role in CNS immunity and synaptic remodeling.

The classical M1/M2 framework distinguishes pro-inflammatory (M1-like) microglia, which release cytokines, ROS, and nitric oxide, from anti-inflammatory/neuroprotective (M2-like) microglia, which promote tissue repair, debris clearance, and release trophic factors such as BDNF and IGF-1 [106,107]. Although microglial phenotypes in vivo are more heterogeneous and dynamic, this binary classification remains useful in translational research. This conceptual framework provides a rationale for therapeutic interventions aimed at shifting microglial responses toward neuroprotection. Therapeutic reprogramming strategies—including CSF1R inhibitors, P2X7 antagonists, and TREM2 agonists—are currently being tested in preclinical models and early clinical trials of Alzheimer’s disease, multiple sclerosis, and Parkinson’s disease [105,108]. These approaches aim to shift the balance towards protective microglial responses, thereby attenuating neuroinflammation and supporting repair.

Among molecular biomarkers, CSF sTREM2 has emerged as a robust indicator of microglial activation, with elevated levels reported in Alzheimer’s disease and ALS [109,110,111]. TMEM119 remains widely used to identify resident microglia, though its specificity may be reduced under cellular stress conditions [112].

In multiple sclerosis (MS), chronic microglial activation exacerbates demyelination and neurodegeneration by releasing pro-inflammatory cytokines (e.g., TNF-α, IL-1β), chemokines, and reactive oxygen species, while impairing clearance of myelin debris. These maladaptive responses hinder remyelination and accelerate axonal loss. Recent advances have identified Bruton’s tyrosine kinase (BTK) signaling as a critical regulator of microglial activation. Pharmacological BTK inhibitors (e.g., evobrutinib, tolebrutinib) are under investigation for their capacity to attenuate microglial-driven inflammation and to promote a shift toward reparative phenotypes. Early clinical trials suggest that BTK inhibitors may reduce inflammatory activity and preserve white matter integrity, underscoring their potential as novel modulators of microglial function in MS [113,114,115].

**Figure 4:**
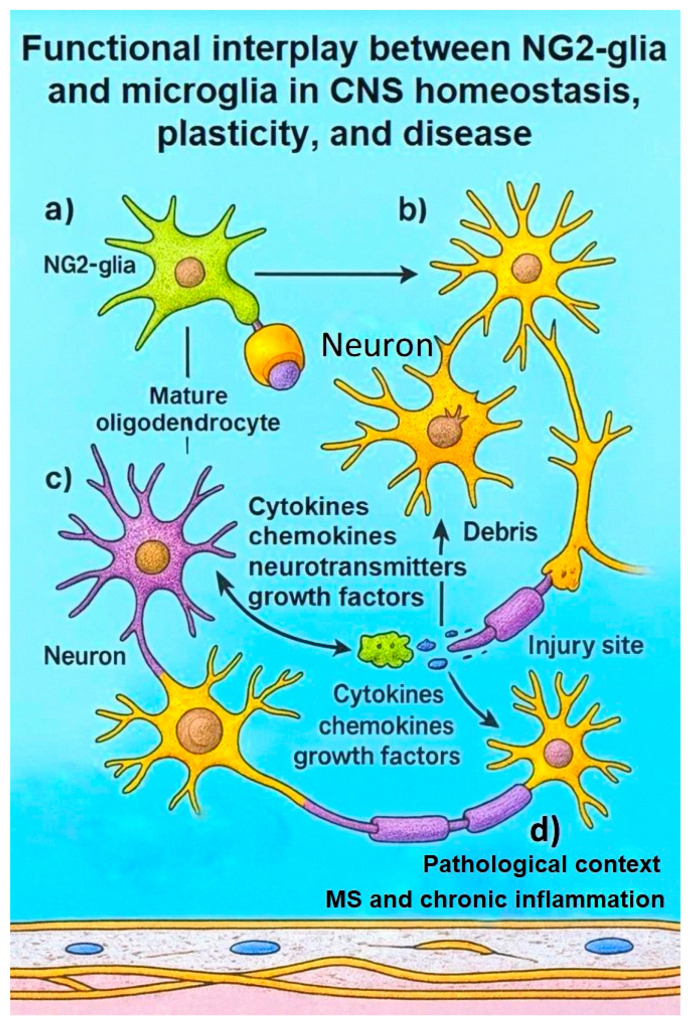
Functional interplay between NG2-glia and microglia in CNS homeostasis, plasticity, and disease. (a) Crosstalk under physiological conditions. (b) Involvement in injury response and tissue repair. (c) Cytokine-, chemokine-, and neurotransmitter-mediated signaling pathways driving neuroinflammation. (d) Pathological activation and maladaptive interactions in demyelinating conditions such as multiple sclerosis (MS) and chronic inflammation.

Although NG2-glia are not conventional immune effectors, they engage in extensive crosstalk with microglia. They can respond to pro-inflammatory cues by altering their proliferation, differentiation, and secretion of trophic factors, and can reciprocally influence microglial activation states [116,117]. This bidirectional interaction is particularly relevant in demyelinating conditions such as multiple sclerosis, where maladaptive NG2-glia responses and microglial dysregulation synergistically impair remyelination and exacerbate neurodegeneration [116,117]. Pathological crosstalk between these cell types fosters chronic inflammation and demyelination (Figure 4).

#### 2.3.4. Intercellular Interactions and Neurovascular Coupling

NG2-glia and microglia are integral components of highly interconnected glial networks, engaging in multidirectional communication with astrocytes, neurons, endothelial cells, and other vascular elements to preserve CNS homeostasis. NG2-glia, often located in close proximity to blood vessels, are strategically positioned to influence BBB integrity, perivascular signaling, and local metabolic exchange [118,119,120,121,122,123]. Through their perivascular endfeet and interactions with astrocytic processes, they participate in nutrient delivery and regulation of extracellular ionic balance. Microglia, in turn, contribute to neurovascular coupling by detecting neuronal activity-related metabolic changes and signaling to vascular elements. They coordinate adaptive vascular responses in cooperation with astrocytes and pericytes, and under pathological conditions, they can modulate BBB permeability, upregulate adhesion molecules on endothelial cells, and recruit peripheral immune cells. This dual ability to maintain vascular stability under physiological conditions and rapidly alter vascular function in disease states underscores the importance of both NG2-glia and microglia as central mediators of CNS perfusion and immune surveillance [104,123].

#### 2.3.5. Pathological Roles and Therapeutic Relevance

Aberrant activity of NG2-glia and microglia is increasingly recognized as a driver of pathology in neurodegenerative and demyelinating disorders such as AD, PD, and MS [97,124]. Under these conditions, chronic microglial overactivation sustains a pro-inflammatory milieu characterized by the release of reactive oxygen and nitrogen species, excitotoxic mediators, and proteases, which collectively exacerbate neuronal injury and synaptic loss. Simultaneously, NG2-glia dysfunction—manifesting as impaired proliferation, migration, or differentiation into myelinating oligodendrocytes—compromises remyelination and deprives neurons of metabolic and structural support.

OPCs are classically identified by co-expression of NG2/CSPG4 and PDGFRα, markers that remain the gold standard for defining this lineage [124]. Recent work also implicates OPC dysfunction in aging and Alzheimer’s disease, further supporting their relevance as both mechanistic and diagnostic targets [125,126,127,128,129].

Therapeutically, both cell populations represent promising but distinct targets. Strategies aimed at promoting NG2-glia lineage progression and enhancing oligodendrogenesis hold potential for restoring white matter integrity and improving axonal conduction. Conversely, immunomodulatory approaches that reprogram microglia toward a neuroprotective, anti-inflammatory phenotype may attenuate ongoing tissue damage and promote repair. Emerging cutting-edge modalities—from small-molecule inhibitors and monoclonal antibodies to epigenetic modulators and next-generation gene-editing platforms—are being strategically developed to fine-tune glial cell activity, opening avenues for synergistic therapies that simultaneously suppress inflammation and enhance remyelination [87,89,119,129].

### 2.4. Translational Perspectives and Future Directions

The expanding recognition of glial cells as active, multifunctional regulators of the CNS function marks a transformative shift in neuroscience. Once regarded primarily as passive structural and metabolic supporters, glial cells are now acknowledged as dynamic participants in synaptic transmission, neurovascular coupling, neural plasticity, and neuroinflammatory processes. This paradigm shift not only redefines our understanding of brain physiology but also positions glia as viable, high-value therapeutic targets. Bridging the gap between basic cellular neurobiology and clinical translation will be essential for harnessing their full therapeutic potential. For example, recent translational work on oligodendrocytes has demonstrated that remyelination can be promoted by compounds such as clemastine fumarate, as well as by gene therapy approaches that enhance OPC differentiation [63,64]. Similarly, microglial biology is increasingly viewed through the M1/M2 framework, with therapeutic strategies targeting CSF1R, P2X7, or TREM2 to reprogram microglial activation toward neuroprotection [106,107,116,118].

#### 2.4.1. Clinical and Therapeutic Implications

**Figure 5:**
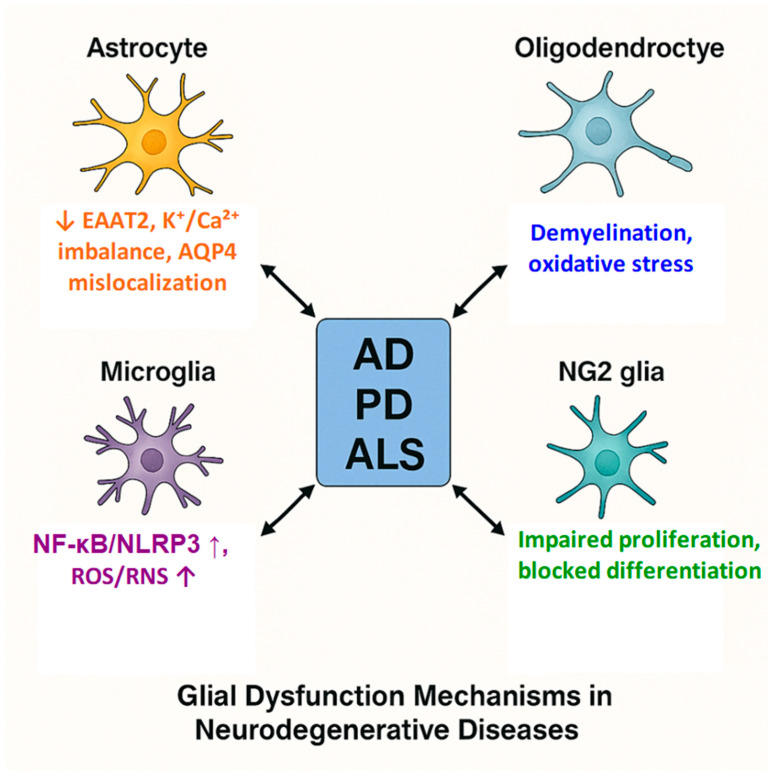
Mechanistic pathways of glial dysfunction in neurodegenerative diseases. Astrocytes: Downregulation of EAAT2 (impaired glutamate clearance), ionic imbalance (K^+^, Ca^2+^), aquaporin-4 (AQP4) mislocalization, altered Ca^2+^ signaling, and astrogliosis disrupt excitatory balance and neurovascular coupling. Oligodendrocytes: Demyelination, oxidative stress, and metabolic disconnection from axons impair myelin integrity and axonal support. Microglia: Chronic NF-κB/NLRP3 activation, excessive ROS/RNS production, impaired proteostasis of misfolded proteins (Aβ, α-synuclein), and pathological synaptic stripping amplify neuroinflammation and neuronal injury. NG2-glia (OPCs): Impaired proliferation and blocked differentiation, often linked to dysregulated Wnt/β-catenin signaling, limit remyelination and regeneration. Upward (↑) and downward (↓) arrows denote molecular upregulation and downregulation, respectively.

Among all glial subtypes, microglia have emerged as especially attractive therapeutic targets due to their central roles in innate immune surveillance, synaptic pruning, and orchestration of inflammatory responses in both acute and chronic CNS conditions. Pharmacological strategies aimed at reprogramming microglia from pro-inflammatory (M1-like) to neuroprotective (M2-like) states are under intensive investigation. Recent multiomic studies emphasize microglial heterogeneity and context-dependent phenotypes, underscoring the need for cell-type-specific therapeutic approaches [130,131,132]. Preclinical work has demonstrated the feasibility of small-molecule inhibitors, monoclonal antibodies, and CRISPR–Cas9-mediated gene editing in modulating microglial activity in models of AD, MS, PD, and traumatic brain injury [67]. Oligodendrocytes and NG2-glia represent promising targets for remyelination therapies. Single-cell transcriptomic profiling has recently revealed previously unrecognized heterogeneity among glial progenitors, providing novel insights into their roles in remyelination and network plasticity [133]. Interventions designed to stimulate oligodendrocyte progenitor proliferation, migration, and maturation-often via modulation of Wnt/β-catenin or PI3K–Akt–mTOR signaling pathways-hold potential for restoring white matter integrity and axonal conduction in MS, leukodystrophies, and spinal cord injury. Astrocytes, once viewed primarily as metabolic supporters, are now appreciated for their context-dependent roles—offering neuroprotection via metabolic and antioxidant support under physiological conditions, but potentially contributing to excitotoxicity, BBB breakdown, and chronic neuroinflammation when dysregulated. Therapies aimed at modulating astrocytic gliotransmitter release, calcium signaling, or gap junction coupling show early promise in preclinical models of epilepsy, ischemic stroke, and mood disorders [65,70,102]. Collectively, these findings position glial cells not as passive bystanders, but as integrative control hubs in CNS homeostasis and pathology—making them compelling targets for next-generation interventions. Importantly, glial dysfunction is not limited to a generic ‘loss of homeostasis’ but reflects cell-type-specific processes. In astrocytes, downregulation of EAAT2, ionic imbalance, and aquaporin-4 mislocalization disrupt excitatory balance and glymphatic clearance; in oligodendrocytes, demyelination and oxidative stress compromise axonal support; in microglia, NF-κB/NLRP3-driven inflammatory signaling and excessive ROS/RNS production amplify neurotoxicity; and in NG2-glia, impaired proliferation and differentiation limit remyelination capacity (Figure 5).

#### 2.4.2. Strategic Research Priorities

Recent preclinical studies underscore the translational feasibility of precision glia-targeted therapies, as shown in the following examples: Microglia reprogramming: use of CSF1R inhibitors to restore homeostatic microglial phenotypes [105]. Oligodendrocyte regeneration: Wnt/β-catenin pathway modulation to enhance remyelination following demyelinating injury. Astrocyte-targeted genome editing: CRISPR–Cas9 strategies directed at connexin-43 or glutamate transporter genes to stabilize synaptic activity in epilepsy and ischemia models. To unlock the full therapeutic potential of glial biology, the following strategic directions are recommended:

1. Advanced imaging and real-time functional mapping (employ super-resolution microscopy, two-photon in vivo imaging, and optogenetic tools to visualize glial dynamics and neuron–glia interactions with subcellular precision in real time) [134].
2. Genetic and molecular dissection (use single-cell multiomics, lineage tracing, and CRISPR-based genome engineering to resolve subtype-specific functions, plasticity states, and disease-associated transcriptional signatures) [75].
3. Computational and systems neuroscience integration (develop multi-scale computational models to simulate neuron–glia network behavior, predict system-level outcomes of pharmacological modulation, and identify optimal therapeutic targets).
4. Interdisciplinary translational frameworks (integrate neuroscience with immunology, systems biology, biomaterials science, and bioengineering to advance glia-based interventions from bench to bedside) [135].

Ultimately, bridging fundamental discoveries with clinical translation will require rigorous validation in disease-relevant models and early-phase clinical trials designed to evaluate safety, target engagement, and efficacy in human CNS disorders. The next decade of translational neurobiology is poised to see glia-targeted therapeutics emerge as a cornerstone of precision medicine in neurology and psychiatry.

### 2.5. Ependymal Cells

Ependymal cells (ependymocytes) are a morphologically distinct and functionally versatile glial cell population that lines the ventricular system of the brain and the central canal of the spinal cord [135,136]. Organized into a simple cuboidal-to-columnar epithelium termed the ependymal layer, they act as a dynamic interface between CSF and brain parenchyma, actively participating in CNS homeostasis [137]. New transcriptomic evidence indicates that glial populations undergo dynamic transcriptional changes across sleep–wake cycles, further linking their activity to circadian regulation of CNS homeostasis [138]. Once regarded merely as a passive epithelial lining, ependymal cells are now recognized as mechanosensory, secretory, and regulatory hubs, influencing CSF dynamics, neurogenesis, and potentially neuronal communication [136,137].

#### 2.5.1. Structure and Localization

Ependymocytes exhibit cuboidal or columnar morphology and are characterized by dense apical arrays of motile cilia and microvilli projecting into the ventricular lumen [100]. The coordinated beating of motile cilia generates laminar CSF flow, while microvilli increase absorptive and secretory surface area [137]. Intercellular adherens junctions maintain epithelial integrity, yet the absence of tight junctions confers selective permeability, enabling regulated exchange of solutes, metabolites, and signaling molecules between CSF and periventricular tissue [98,137,139,140]. Recent single-cell transcriptomic analyses have revealed molecular heterogeneity within ependymal populations, suggesting the existence of region-specific subtypes with specialized functions.

FoxJ1 is the canonical transcription factor defining ependymal cell fate and ciliary function, and FoxJ1^+^ ependymal populations persist throughout the human lifespan [141]. Importantly, heterozygous FOXJ1 mutations have been directly linked to hydrocephalus, establishing a translational bridge between basic ependymal biology and clinical pathology [142].

#### 2.5.2. Role in CSF Dynamics

**Figure 6:**
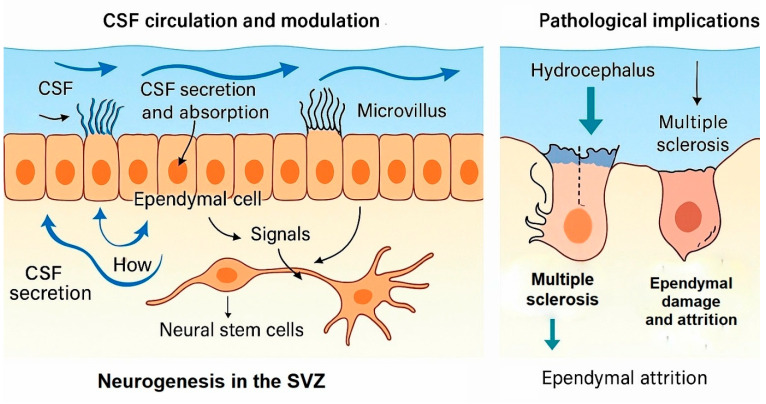
Schematic representation of cerebrospinal fluid (CSF) circulation and modulation by ependymal cells. Motile cilia on ependymal cells generate directional CSF flow through the ventricular system and central canal, while microvilli facilitate absorption and secretion of solutes. Localized interactions with the choroid plexus contribute to CSF composition, and periventricular contact with neural stem cells supports neurogenesis in the subventricular zone (SVZ).

While the choroid plexus remains the principal producer of CSF, ependymal cells are critical for flow regulation, composition modulation, and homeostatic balance [136,143,144,145]. Coordinated ciliary beating ensures directional flow along the ventricular axis, facilitating nutrient distribution, waste removal, and intracranial pressure regulation [139,140]. Mechanosensory proteins such as polycystin-1/2 and Piezo1/2 in ciliary membranes detect shear stress and fluid velocity, potentially modulating downstream signaling cascades that influence both CSF secretion and local cellular responses (Figure 6).

#### 2.5.3. Neurogenesis and the Neural Stem Cell Niche

Ependymal cells are essential components of the subventricular zone (SVZ) neurogenic niche [145,146]. They provide physical scaffolding for neural stem cells (NSCs) and secrete molecular cues—such as fibroblast growth factor-2 (FGF2), epidermal growth factor (EGF), and extracellular matrix proteins—that regulate NSC proliferation, differentiation, and migration [143,144,145]. This interaction facilitates lifelong neurogenesis and is implicated in structural brain plasticity. Disruption of ependymal–NSC communication can impair regeneration after injury and contribute to cognitive decline.

#### 2.5.4. Sensory and Signaling Functions

Strategically positioned at the CSF–brain interface, ependymal cells act as chemo- and mechanosensors, detecting changes in ion concentrations, neurotransmitter levels, osmolarity, and pH in the CSF [105]. Through paracrine signaling, they can influence nearby astrocytes, microglia, and neurons, thereby integrating ventricular milieu monitoring with broader neurophysiological regulation [136,144]. This sensory role is increasingly recognized as a potential mechanism by which systemic changes—such as inflammation or metabolic stress—can influence CNS function.

#### 2.5.5. Pathological Implications

Ependymal dysfunction contributes to a range of neurological diseases. Ciliary dyskinesia impairs CSF circulation, causing non-communicating hydrocephalus and increased intracranial pressure [147,148,149,150]. In MS, structural loss of ependymal cells can alter CSF–brain barrier permeability, facilitating immune cell infiltration and neuroinflammation [151,152]. Age-related ependymal attrition is associated with reduced ciliary motility, impaired waste clearance, and altered neurogenesis, potentially contributing to neurodegenerative processes in AD and PD.

#### 2.5.6. Research and Therapeutic Potential

Due to their accessibility, plasticity, and integration within neurogenic niches, ependymal cells are emerging as therapeutic targets [153]. Potential strategies include the following:

- Ciliary function restoration via gene therapy or pharmacological modulation to improve CSF dynamics.
- Barrier reinforcement to limit neuroinflammation in demyelinating disorders.
- Neurogenic niche activation through targeted delivery of growth factors or bioengineered scaffolds to enhance endogenous regeneration [134,153].

The integration of single-cell multiomics, high-resolution live imaging, and biomaterial-based delivery systems is expected to accelerate the translation of ependymal cell biology into regenerative medicine, potentially transforming the management of hydrocephalus, traumatic CNS injury, and neurodegenerative diseases (Table 1).

## 3. Advanced Imaging and Integrative Methodologies in Glial Research

Recent technological advances have revolutionized our ability to investigate glial cell biology, enabling unprecedented spatial, temporal, and molecular resolution of their roles in the CNS. Cutting-edge imaging, genetic manipulation, computational modeling, and translational strategies are converging to redefine glial research, transforming it from descriptive morphology into a highly mechanistic, systems-level discipline.

### 3.1. Advanced Imaging Technologies

The emergence of state-of-the-art optical imaging modalities—including super-resolution microscopy (e.g., STED, SIM, PALM/STORM), two-photon and three-photon excitation microscopy, and high-speed live-cell fluorescence imaging—has enabled real-time, in situ visualization of glial morphology, motility, and intercellular interactions at nanometer resolution [135]. These techniques capture dynamic glial responses to physiological stimuli (e.g., synaptic activity, vascular changes) and pathological insults (e.g., ischemia, neuroinflammation) at subcellular, synaptic, and circuit levels. Combining these optical tools with genetically encoded reporters (e.g., GCaMP for Ca^2+^ dynamics, phluorin-based synaptic sensors) allows mapping of functional signaling events within intact neural tissue, bridging structural and functional perspectives in glial biology.

### 3.2. Genetic and Molecular Manipulations

The development of precision molecular tools—including CRISPR-Cas9 genome editing, RNA interference, epigenome editing, chemogenetics (DREADDs), and optogenetics—has facilitated targeted manipulation of glial subtypes with cell-type specificity and temporal precision [75]. These approaches allow researchers to dissect distinct glial contributions to synaptic modulation, neuroinflammation, myelination, and neurovascular coupling. Integration with single-cell transcriptomics and proteomics enables the identification of glial subtype-specific biomarkers and druggable pathways. Moreover, combining optogenetic stimulation of astrocytes or microglia with in vivo imaging platforms creates a direct causal link between glial activity patterns and behavioral outcomes in animal models.

### 3.3. Computational and Systems Neuroscience

Computational modeling is becoming a cornerstone of modern glial research, providing the analytical capacity to integrate multimodal datasets—from electrophysiology to imaging and omics—into predictive, mechanistic models. These in silico frameworks simulate glial–neuronal network dynamics, enabling predictions of how perturbations in glial physiology influence higher-order processes such as network excitability, oscillatory activity, and cognitive function [75,134]. Machine learning algorithms applied to large-scale imaging datasets can uncover hidden patterns in glial morphology and activity, guiding hypothesis generation and experimental design.

### 3.4. Interdisciplinary Integration

A holistic understanding of glial biology requires integration of disciplines including neuroscience, immunology, vascular biology, systems biology, and bioengineering [135]. Such collaboration bridges molecular and systems-level data, yielding insights into how glial cells maintain CNS homeostasis, immune surveillance, metabolic support, and structural plasticity. This interdisciplinary approach also supports the translation of basic discoveries into clinical applications, particularly in the context of neurodegenerative and neuroinflammatory diseases.

### 3.5. Translational Neuroscience and Glia-Targeted Therapeutics

Translating basic glial research into next-generation therapies is an emerging frontier in neurotherapeutics. Pathological glial remodeling is now recognized as a primary driver in conditions such as AD, MS, gliomas, epilepsy, and traumatic brain injury. Strategies include the following:

- Modulating microglial–neuronal crosstalk with CX3CR1 agonists or attenuating chronic neuroinflammation via CSF1R inhibitors.
- Enhancing remyelination through transplantation of iPSC-derived oligodendrocytes or pharmacological activation of oligodendrocyte progenitor cells.
- Reprogramming astrocyte reactivity to restore neurovascular coupling and reduce excitotoxicity in stroke and epilepsy models.

**Figure 7:**
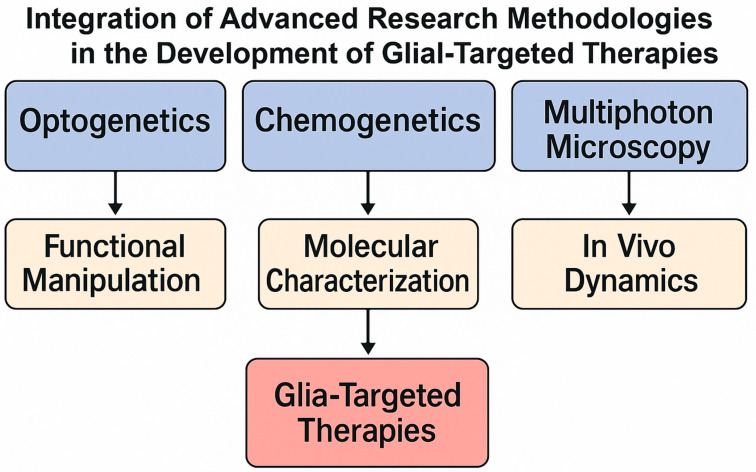
Integration of advanced research methodologies in the development of glial-targeted therapies. Optogenetics and chemogenetics enable precise functional manipulation of glial cells. Transcriptomic profiling provides molecular characterization, while multiphoton microscopy allows dynamic in vivo imaging. Collectively, these complementary approaches converge to accelerate the development of glia-targeted therapeutic strategies.

These therapeutic concepts are moving toward clinical translation, with ongoing early-phase trials reporting encouraging safety and efficacy signals. Emerging tools such as gene editing, optogenetics, and chemogenetics have enabled unprecedented precision in dissecting glial cell functions directly in vivo (Figure 7). These approaches not only provide mechanistic insights into glia–neuron interactions but also open translational avenues for cell-type-specific therapeutic interventions.

The future of CNS therapy will increasingly depend on targeting glial plasticity and repair mechanisms, rather than solely focusing on neurons. Pharmacological agents regulating glial activation states, neuroimmune signaling, and remyelination hold promise for disease modification. Furthermore, stem cell-based and gene therapy approaches aimed at replacing or rejuvenating dysfunctional glia could transform the treatment landscape for neurodegeneration, neuroinflammation, and CNS injury. Glial cells are therefore no longer viewed as passive structural supporters but as dynamic regulators of CNS homeostasis and pathology, positioning them as promising therapeutic targets for next-generation interventions. Importantly, by integrating recent original research articles with the established review literature, this manuscript provides a balanced synthesis of astrocytic contributions to neurological disorders. Incorporation of findings from transcriptomic profiling, mitochondrial dysfunction studies, and astrocyte-derived protein secretion analyses ensures that the discussion reflects both mechanistic depth and translational relevance.

## 4. Conclusions

Over the past decade, glial biology has undergone a paradigm shift-from being perceived as a passive support system to emerging as a central, dynamic regulator of CNS function and pathology. This review underscores several meaningful breakthroughs that redefine the role of glial cells in brain health and disease:

1. Recognition of glial heterogeneity and plasticity as a driver of neural network adaptation—Single-cell multiomics and high-resolution in vivo imaging have revealed unprecedented diversity within astrocytes, oligodendrocytes, microglia, NG2-glia, and ependymal cells, uncovering their context-dependent functions in synaptic modulation, neurovascular coupling, and immune regulation.
2. Identification of glia-specific molecular targets with translational potential—novel therapeutic strategies now focus on modulating glutamate transporters in astrocytes, promoting remyelination via OPC differentiation, reprogramming microglia toward neuroprotective phenotypes, and restoring glymphatic clearance through aquaporin-4 polarity correction.
3. Demonstration of glial roles in cognition, plasticity, and repair—evidence now shows that activity-dependent myelination, astrocyte–neuron lactate shuttling, and microglia-mediated synaptic remodeling are integral to learning, memory, and post-injury regeneration.
4. Integration of glial biology into precision medicine frameworks—CRISPR–Cas9 editing, chemogenetic modulation, and bioengineered scaffolds targeting glia are entering preclinical and early-phase clinical trials, bridging basic discoveries with therapeutic application in neurodegenerative, neuroinflammatory, and psychiatric disorders.
5. Emergence of glial biomarkers for early diagnosis and disease monitoring—circulating or CSF-derived glial markers (e.g., GFAP, sTREM2) are now being evaluated as prognostic tools, enabling patient stratification and therapy tracking.

By consolidating these advances, it becomes clear that glia-targeted interventions—from small molecules and gene therapies to regenerative medicine—will likely become a cornerstone of next-generation CNS therapeutics. Fully harnessing glial plasticity and heterogeneity will require integrating molecular profiling, advanced imaging, and systems biology into translational pipelines, ultimately transforming the prevention and treatment of brain disorders.

## Figures and Tables

**Table 1:** Biomarkers of major glial cell types and their clinical significance. The table summarizes molecular and immunological biomarkers currently used to identify glial cell populations, with emphasis on their diagnostic and translational relevance in neurological disorders.

Glial Cell Type	Molecular Biomarkers	Principal Physiological Functions	Disease-Associated Alterations	Translational/Diagnostic Relevance
Astrocytes	GFAP, S100β, AQP4, EAAT1/2	Ion homeostasis, neurotransmitter clearance, metabolic support, BBB regulation, immune surveillance	Reactive astrogliosis, impaired glutamate uptake, AQP4 mislocalization, neuroinflammation	GFAP (CSF/plasma)—biomarker of AD progression [48]; AQP4-IgG—diagnostic of NMOSD [49]
Oligodendrocytes	MBP, MOG, PLP1, CNPase	Myelination, axonal insulation, metabolic support	Demyelination (MS, leukodystrophies), oxidative stress, impaired coupling	CSF MBP—biomarker of demyelination [82]; MOG-IgG—defines MOGAD [80,81]
NG2-glia (OPCs)	NG2/CSPG4, PDGFRα, Olig2	Proliferation, differentiation, remyelination, synaptic modulation	Blocked differentiation, maladaptive responses in demyelination	Canonical identifiers: NG2/CSPG4, PDGFRα [125]; OPC dysfunction in aging and AD [126,128]
Microglia	Iba1, TMEM119, CX3CR1	Immune surveillance, synaptic pruning, phagocytosis	Chronic activation, NF-κB/NLRP3 signaling, oxidative stress	CSF sTREM2—biomarker of microglial activation in AD, ALS [109,111]; TMEM119—resident microglial marker (limitations) [112]
Ependymal cells	Vimentin, CD24, FoxJ1	CSF circulation, barrier function, support of neurogenic niches	Impaired CSF flow, barrier breakdown, hydrocephalus	FoxJ1—canonical marker [141]; FOXJ1 mutations—linked to hydrocephalus [142]

Major glial cell types in the CNS, their molecular biomarkers, physiological functions, disease-associated alterations, and translational/diagnostic relevance. References are provided in Vancouver style to support each entry. Abbreviations: GFAP—glial fibrillary acidic protein; S100β—S100 calcium-binding protein β; AQP4—aquaporin-4; EAAT—excitatory amino acid transporter; MBP—myelin basic protein; MOG—myelin oligodendrocyte glycoprotein; PLP1—proteolipid protein 1; CNPase—2′,3′-cyclic-nucleotide 3′-phosphodiesterase; NG2—neural/glial antigen 2; PDGFRα—platelet-derived growth factor alpha; Olig2—oligodendrocyte transcription factor 2; Iba1—ionized calcium-binding adapter molecule 1; CX3CR1—C-X3-C motif chemokine receptor 1; TMEM119—transmembrane protein 119; CD24—cluster of differentiation 24; FoxJ1—forkhead box protein J1.

## Data Availability

No new data were created or analyzed in this study. Data sharing is not applicable to this article.
